# Immersive virtual reality: An effective strategy for reducing stress in young adults

**DOI:** 10.1177/03080226231165644

**Published:** 2023-04-01

**Authors:** Sarah McGarry, Ashleigh Brown, Miranda Gardner, Caitlan Plowright, Renee Skou, Craig Thompson

**Affiliations:** Curtin School of Allied Health, Curtin University, Perth, WA, Australia

**Keywords:** Virtual reality, stress, relaxation, immersive, young adults

## Abstract

**Background:**

Psychological stress is ubiquitous in young adults, but traditional relaxation strategies such as mindfulness or meditation are not consistently utilised. Immersive virtual reality (VR) is being increasingly used in mental health contexts with improved clinical outcomes. However, there is limited research exploring the perceived usefulness of VR for individuals without a mental health diagnosis.

**Aim:**

To identify if an immersive VR experience was effective in reducing stress for young adults and to explore the experience of the participants.

**Methods:**

Twenty-one young adults aged 18–25 years participated in an immersive VR experience. Heart rate was measured pre- and post-test to objectively establish the efficacy of VR to facilitate relaxation. Participants were invited to participate in a semi-structured interview (*n* = 18) to gain a rich understanding of their experience.

**Results:**

Four themes emerged from analysis of the interviews: *I felt relaxed and calm, It was time-efficient and easy to use, It took me to another place and It was different than I thought it would be*. Heart rate reduced during the immersive VR experience.

**Conclusion:**

These findings suggest that immersive VR can reduce psychological stress, and it is perceived by young adults as a useful and time-effective strategy to reduce psychological stress.

## Introduction

Psychological stress is a disturbance in one’s homeostasis that produces physical, mental and emotional responses, caused by stressors that are perceived by the person to exceed their adaptive capacity (Sood et al., 2013). Psychological stress causes continuous changes in the autonomic nervous system, causing an alteration in heart rate (Furutani et al., 2011). Young adults between 18 and 25 years reported the highest level of stress and the lowest level of psychological well-being (Australian Psychological Society, 2015). Sources of elevated psychological stress in young adults can be due to a combination of financial, academic and social pressures (Schut et al., 2016). Psychological stress can also negatively impact academic performance and social engagement (Hamaideh, 2011) and can result in maladaptive coping strategies such as smoking and substance misuse (Salzer, 2012). Additionally, stress can impact the level of engagement young adults have in meaningful activities and their ability to achieve occupational balance (Chang et al., 2017).

Stress management strategies are crucial in managing levels of distress to reduce the likelihood of developing a mental illness; many young adults do not utilise positive coping strategies or seek help until reaching a crisis (Deasy et al., 2014). Relaxation techniques are used to calm the sensory system and regulate the body’s hormonal levels and neurological state to return to homeostasis (Deasy et al., 2014). While stress management techniques such as relaxation and mindfulness are useful approaches to managing stress and anxiety, they are difficult and time-consuming to master effectively (Repetto et al., 2009). Young adults are time poor and many coordinate full-time studies with part-time employment as well as other domestic and social commitments (Crozier et al., 2008). This may contribute to young adults tending not to engage in stress management initiatives, and literature highlights a need for more appropriate strategies for managing distress in these populations (Chiauzzi et al., 2008). Furthermore, engaging in some form of relaxation, such as mindfulness, especially when combined with everyday occupations, is beneficial to the well-being and self-care of young adults, especially in the context of the stresses involved in higher education (Stew, 2011). Many young adults show their preference and comfortability to utilising technology, as well as possessing superior information technology skills to other populations (Akçayır et al., 2016). In an effort to target young adults, health professionals have started to incorporate information technology into stress management interventions (Chiauzzi et al., 2008). Of particular interest is the ongoing development of virtual reality (VR) technology (Dascal et al., 2017).

VR platforms are becoming increasingly affordable and accessible as an intervention, and there has been an increasing amount of literature evaluating the effectiveness of immersive VR for a range of mental health disorders (Valmaggia et al., 2016). It has been recognised that immersive VR effectively reduces stress levels in individuals through the use of natural virtual environments (Anderson et al., 2017). The reduction in stress levels was evidenced by a reduction in heart rate and other measures (Anderson et al., 2017). This reduction in heart rate is indicative of a sense of calm and increased relaxation. A change in heart rate can be used as an objective measure of stress, as demonstrated in numerous research studies (Fominykh et al., 2006).

There is emerging evidence supporting the use of immersive VR to manage psychological stress, which with the increasing availability and affordability of the technology makes it a cost-effective option (Valmaggia et al., 2016). Further research on the impact of immersive VR is required to support its use for young adults who experience psychological stress. Preliminary research has indicated that many participants find the experience of immersive VR useful; however, more in-depth accounts of this experience would be beneficial to inform clinical practice and stress management strategies available to young adults (Dascal et al., 2017). The primary aim of the present study was to explore the experience of immersive VR for reducing perceived stress levels, from the perspective of young adults. The secondary aim of this study was to explore if there was a change in heart rate before and after the use of immersive VR.

## Methods

### Research design

This study employed a convergent parallel mixed-method approach in order to facilitate the collection of data from multiple perspectives. In doing so a greater understanding of the research topic can be explored. The purpose of utilising this mixed methods approach was complementarity (Greene et al., 1989). This facilitated facets of the research phenomenon to be measured to allow for a richer and more holistic understanding of immersive VR. The qualitative aspect was the priority of this study. The qualitative component of this study employed an interpretative phenomenological approach to collect and analyse data (Smith et al., 2009). A phenomenological study design was considered a suitable approach as it enabled researchers to gain insight into the experience of using immersive VR (Smith et al., 2009).

The quantitative component of the study comprised a pre- and post-test measure of the heart rate of the participants. Researchers measured each participant’s heart rate before commencing the immersive VR experience (time-point 1) and then repeated the measurement upon completion of the 15-minute immersive VR session (time-point 2).

In this study, the participants’ experience of engaging in the immersive VR was explored using semi-structured interviews. This was completed with a researcher and participants who elected to participate in an interview, following the VR session. The interviews were recorded and provided comprehensive data which contributed to a thorough understanding of the participants’ experiences (Liamputtong, 2013). Integration of the qualitative and quantitative data occurred at the interpretation and reporting level via narrative description (Fetters et al., 2013). Quantitative data collected were examined and integrated with qualitative data to explain and validate all results. The process of combining the quantitative data and the semi-structured interview data in the mixed-method design triangulated results and contributes to increased credibility (Srnka & Koeszegi, 2007).

### Participants

Young adults between the ages of 18 and 25 years who were living within the Perth metropolitan area and self-reported experiencing psychological stress were invited to participate in this study. A total of 21 young adults (16 females, 5 males; *M* age = 23.04; SD = 1.6) participated, with 18 completing both aspects of the study. One participant (1 female; age = 23) elected not to complete the VR session or post-test heart rate measurement due to reported visual disturbances while wearing the VR goggles. Two participants (2 females; *M* age = 25.5; SD = 0.5) completed the VR session but did not complete the pre-test and post-test heart rate measurements due to the heart rate monitor chest strap malfunctioning. Participants were recruited via advertisements shared on social media platforms. A snowball sampling method was used to recruit participants which allowed access to individuals who shared the characteristics required for the study.

### Materials

#### Protocol

Researchers created a protocol (Table 1) for the VR session that was informed by current literature and collaboration with an occupational therapist experienced in the use of VR in practice. The protocol detailed the length of the session, the equipment used, and instructions provided.

**Table 1.** VR research protocol.

Step	Description
1.	At the beginning of each data collection session, participants were seated and provided with a consent form and information form to read and sign.
2.	Researchers verbally summarised the purpose and conditions of the study as well as the possible adverse events which may occur, for example, motion sickness, headaches, disorientation, increased anxiety, discomfort wearing the VR equipment and claustrophobia.
3.	Participant put on the heart rate monitor around their chest. After the heart rate monitor is in place, the researchers orientate them to the VR hand controller. - The controller appeared as a laser pointer when the VR goggles are on. - To select the program, participants held the laser pointer over the Nature Treks app and pressed the trigger on the back of the controller. - When the program has been selected, the participant used the trigger to move around within the scene by holding it in to ‘walk’. - The touch surface acted as a mouse, allowing the participant to change direction within the scene. - The program allowed the user create items within the environment. By clicking the bottom of the touch surface, bringing up options and clicking the top of the touch surface actioned the option within the environment. - To exit the environment, the participant can click the back or home button at any time.
4.	When then the controller was explained to the participant, the VR goggles will be turned on and the participant was guided to choose the Nature Treks beach scene. The participant was told that they had 15 minutes with the VR goggles and that their heart rate was being recorded at the start of the session and at the end of the session.
5.	After the session, the participant was asked to take off the heart rate monitor; this was then cleaned with a disinfectant before the next participant used it. The goggles and hand controller were also wiped down before the next participant. The goggles were put on charge between participants to allow for sufficient battery for trial.
6.	Once the session was completed, the participant was invited to participate in a semi-structured interview to gain an understanding of their VR experience.

#### Oculus Go

A portable Oculus Go VR (https://www.oculus.com/) system was used to deliver the session to participants. The Oculus Go consists of a portable headset, hand controller, charging port and transport bag. The portable headset is designed to be used in any environment. Researchers ensured the environment was conducive to a relaxing experience, and all clutter was removed from the room. Researchers sterilised the headset and controller using antibacterial wipes and hand sanitiser between each session.

#### Blue Ocean by Nature Treks VR/Greener Games – VR program on Oculus Go

Blue Ocean (https://greenergames.net/) is a relaxation VR application which was delivered to the participants. The program utilises a soundscape and allowed participants to watch and react to their virtual environment of a beach scene using the hand controller to interact with the environment. The program was designed to induce relaxation and a specific emotional state through the use of immersive colour and sounds.

#### CooSpo H603B by SDK – Chest strap heart rate monitor

The CooSpo H603B (http://www.coospo.com/) was used to measure participants’ heart rates. Chest strap heart rate monitors are more reliable and valid than wrist-worn heart rate monitors as they function similarly to an electrocardiogram, in which they detect cardiac electrical activity (Gillinov et al., 2017). This provided reliable and accurate measurements of heart rate. The strap was worn as per the CooSpo guidelines. The strap was moistened with water on a paper towel prior to wearing for each participant, the strap was placed on the skin comfortably under the rib cage and adjusted to fit securely on the participant, ensuring the moistened electrodes were firmly against the skin. The electrodes were cleaned after every participant using antibacterial wipes. Placement of the electrodes and chest strap in the described position allowed for accurate measurements of heart rate. Heart rate data were measured through a mobile phone application (Polar Beat, https://www.polar.com/) which is compatible with the CooSpo, the pre- and post-data points were measured for 30 seconds, and the average was recorded for each.

#### Interview

Semi-structured interviews were conducted at the end of the immersive VR experience, aiming to explore the participants’ expectations and experience of using the VR as well as their experience of psychological stress in their everyday life. The interview guideline (Table 2) was developed using open-ended questions and prompts which allowed the researchers to ensure some control over the questions asked whilst still allowing for emerging themes to develop and be further explored (Smith et al., 2009). Interview topics included participants’ previous experience with VR platforms, their experience of stress in everyday life, expectations and experience of the VR session, and their perceptions of the usefulness of VR as a tool to manage their own stress levels. The interviews were conducted and recorded using a Dictaphone in the privacy of a quiet room at Curtin University. A diary was used to record emerging themes.

**Table 2.** Semi-structured interview guide.

Interview guide
Have you had any experience with VR platforms before? What did you expect before this VR experience? Could you describe your experience of stress in everyday life? Can you tell me how you found the VR experience? How did the VR experience impact your emotions and how you felt at the time? What are your perceptions of the usefulness of VR as a tool to manage stress levels?

To increase consistency across interviews, the researchers conducted mock interviews and discussed strategies for prompting or rephrasing questions to participants. This ensured that the researchers were adequately prepared with the skills to follow the developed interview guideline at a suitable pace while remaining flexible and responsive to the interviewee (Lysack et al., 2006). The audio files were transferred to a password-protected drive for transcription and analysis.

### Data collection

Participants were recruited and informed written consent was obtained prior to involvement in all components of the study. Participants engaged in the immersive VR experience at Curtin University following the developed protocol. The participants were asked to wear the CooSpo chest strap heart rate monitor, which had been moistened and cleaned, and the resting heart rate measurement was taken with the participant in a seated position. The participants then undertook the 15-minute VR session. On completion of the VR experience, another measurement of the participant’s heart rate was taken. Directly after the VR experience, the participants were invited to participate in a semi-structured interview, which was recorded. The VR experience and interviews took place on campus at Curtin University, using comfortable, private rooms free from distraction. Reflexivity was incorporated into the research process through the use of a diary by the researchers to strengthen the quality of the data collected (Lysack et al., 2006).

### Data analysis

#### Quantitative analysis

The data were analysed using the Statistical Package for the Social Sciences (SPSS) program version 25 (IBM Corp., 2017). The effect of the VR experience on relaxation was measured using the difference between the pre- and post-test scores measuring heart rate. A Kolmogorov–Smirnov normality test was used to determine whether the data were normally distributed. The data were noted to be normally distributed; therefore, a *t*-test was conducted to examine differences between the pre- and post-test heart rate measures.

#### Qualitative analysis

Researchers recorded emergent themes in a diary at the time of data collection and throughout the transcription of interviews, enhancing the trustworthiness of the data through researcher reflexivity (Nowell et al., 2017). Researchers transcribed the semi-structured interviews verbatim and the data were analysed using NVivo software (QSR International Pty Ltd., 2018), which used the transcripts to code and analyse emergent themes (QSR International Pty Ltd., 2018). The researchers followed a structured approach to the analysis of the interviews using interpretive phenomenological analysis increasing the dependability of the study (Nowell et al., 2017). All researchers conducted the following six-step process: (1) Reading and transcribing the data and noting down initial ideas, (2) Generating codes systematically for key features of data and then grouping data relevant to each of the codes, (3) Generating potential themes by collating the codes and sorting data into relevant themes, (4) Reviewing all themes, (5) Naming and defining specified themes and 6) Report writing (Smith et al., 2009). During the process, multiple meetings and discussions occurred between researchers. This facilitated triangulation of the results to enhance credibility of the data and reach consistent internal agreement. The analysis of the interviews allowed the researchers to compose a rich description within the results section and enhance the credibility of the themes.

Voluntary member checking was conducted to check the accuracy of the themes. The member checking steps included researchers sharing the data analysis with participants. No responses were received from the participants.

#### Integration of qualitative and quantitative data

The merging of the qualitative and quantitative data occurred via collaboration and discussion within the research team (Fetters et al., 2013). This process was conducted in face-to-face meetings and continued until a consensus was reached.

### Ethical considerations

Ethical approval was obtained from the Human Research Ethics Committee at Curtin University. (HRE2019-0244) in Perth, Western Australia. Researchers gained informed consent from all participants prior to their involvement in the research. Data were de-identified and stored on a secure password-protected drive. Research complies with National Health and Medical Research Council (NHMRC) guidelines and the Declaration of Helsinki (NHMRC, 2007).

## Results

### Qualitative results

Analysis of the data found four interrelated themes that all contribute to the participants finding the VR experience beneficial to relaxation.

#### ‘I felt relaxed and calm’

All participants reported the VR experience to be relaxing. Furthermore, some talked about how this experience was a mood elevator or discussed how it brought up positive memories. Having the ability to tailor the environment and make it their own was considered by participants to foster feelings of relaxation. Many participants mentioned they felt so relaxed, they felt they were on holiday.

I think that the best thing about the virtual reality was that it was bringing up different memories which weren’t stress-related ones or work-related ones or studying. It was just like when I was on holiday (Participant 16)

Some participants discussed that it took them a few minutes to work out how to interact with the virtual environment, but once they knew what they wanted to do, they felt comfortable enough to let themselves completely relax.

I think for the first part I was bit uptight. Then once I got the hang of it, it was like you could feel yourself slowly relaxing. (Participant 2)

#### ‘It was time-efficient and easy to use’

Participants considered VR to be a time-efficient way to reduce stress. Many participants reported feeling as if they had been in the environment for much longer than the length of the session. Additionally, participants said that the technology was easy to use and very intuitive, allowing them to quickly understand the program and immerse themselves in it.

I felt like I was relaxing for a lot longer than fifteen minutes. (Participant 8)

Participants described this experience as similar to mindfulness and meditation practices. However, the VR was much easier, more engaging and time efficient. Participants claimed that the relaxing and time-efficient nature of this experience allowed them to recharge and refocus.

I think it definitely felt like I was not as stressed if anything it helped my mind to re-focus and concentrate on this one thing. (Participant 4)

I do meditate, here and there. I’d say when you’re meditating you drift off and you come back and you drift off and you come back, whereas this is like you’re in the one mindset (Participant 9)

#### ‘It took me to another place’

In this theme, participants discussed that they found the experience to be immersive and interactive, thereby allowing them to forget about the outside world, feeling that they were in their ‘own little universe’. Being immersed in the virtual world distracted participants from the stressors in their everyday life, as most of the participants reported that during the session they did not think about their stressors. Some participants related the experience of being on holiday. The audio-visual components were highlighted by participants as contributing to the environment feeling realistic. The graphics for the lightning coupled with the sound effects of the thunder and rain made them feel like they were experiencing a thunderstorm.

You sort of forget about everything else when you’re there. So, all the other things that are going on at the moment, you don’t even think about them because you are, sort of, engrossed in this beach. (Participant 10)

Additionally, participants discussed how they enjoyed the interactivity of the program. Being able to individualise the environment according to their personal preferences made them feel as if the program was tailored to them, which made the experience more engaging. Furthermore, participants described having the freedom to choose what they spent their time doing as having a positive impact on their experience.

Once I realised what I liked and what setting I liked, as in what theme, I was then able to just realise that I could sit back and relax. (Participant 7)

#### ‘It was different than I thought it would be’

In discussing this theme, participants described their prior understanding of VR, what they expected from the experience, and how the experience differed. Five of the participants reported that they had previously used some form of VR; none of the participants had used VR for relaxation purposes previously. The majority of participants claimed that they were not sure what to expect from the VR experience. Most participants reported that they were predominantly aware of VR being used in a gaming context.

I thought it would be a lot more basic, I don’t know much about it, from the videos I’ve seen, the games you can play on there, they’ve always been very, very basic. I was really impressed with how the environment worked; the audio was amazing’ (Participant 6)

Some participants expected that they would be passively engaging with the environment, merely watching the visuals. Other participants expected to be actively engaging with the environment, for example, standing, walking or performing physical actions. Being seated was perceived as being beneficial in aiding relaxation.

When asked where they would perceive this technology to be useful to help manage stress in different contexts. Many of the participants mentioned they would like to have access to VR at home to unwind after the day, in the workplace for a relaxing lunch break, or at university to refocus during stressful exam periods. Participants reported that this would be an efficient way to de-stress quickly during periods of heightened stress. However, barriers identified by participants were high cost and limited accessibility.

I guess in certain situations where you can take ten or fifteen minutes even five minutes, whatever, just to escape from everything that’s going on. (Participant 4)

### Quantitative results

Results of the paired *t*-test (*t* = 5.9, *p* = 0.000, *d* = 1.1) indicated that the decrease in heart rate was significant for participants between time-point 1 (*m* = 83.6; SD = 10.6) and time-point 2 (*m* = 72.7; SD = 9.4).

## Discussion

The results of this study suggest that the use of immersive VR facilitates a reduction in psychological stress in young adults. Immersive VR appears to be a time-efficient strategy to elicit relaxation. It is suggested that VR is intuitive and easy-to-use technology which has the ability to take users to another place, distracting them from their stressors. Participants unanimously discussed how they perceived that having access to this technology for a short break during their busy day would allow them to relax, recharge and be more productive. Despite the participant heart rate being significantly lower and supporting the qualitative data, future research is required to truly determine if this reduction in heart rate is due to the VR intervention. Immersive VR is currently being underutilised within the population of interest due to a limited understanding of the technology and awareness of its benefits for relaxation.

The immersive VR experience elicited reported feelings of relaxation from participants which was supported by the reduction in heart rate as measured between time-point 1 and time-point 2. Vivid graphics and sounds within any immersive virtual environment can also contribute to elevated mood and relaxation (Riva et al., 2007). Having the ability to individualise the virtual environment and the autonomy to engage within the environment; however, they pleased, aided in contributing to feelings of relaxation. Tailoring the environment to suit the personal preferences of the user evoked a greater relaxation response than using a generic VR program (Gorini et al., 2010). Additionally, natural VR environments have been shown to enhance relaxation and positive emotions in a time-efficient manner (Anderson et al., 2017).

Relaxation strategies traditionally used to reduce stress levels are meditation and mindfulness (Crane et al., 2014). Although they have a larger evidence base for their efficacy, some people perceive them to be difficult to engage in effectively and too time-consuming to use in a consistent manner (Crane et al., 2014). The participants of the present study were able to achieve a state of relaxation within a 15-minute time period and highlighted the contrast of time spent in comparison to other relaxation strategies that they had utilised in the past. Navarro-Haro et al. (2017) also explored the impact of a brief VR experience (10-minutes) using individuals who regularly practice mindfulness and found that the participants had notably improved emotional and mindfulness states within the short time frame. Furthermore, these participants rated the intervention with high acceptability, demonstrating the perceived usefulness of VR-based mindfulness (Navarro-Haro et al., 2017). Young adults having a strong understanding of information technology contributed to their ability to easily grasp the concept of VR technology. This meant they were able to utilise the relaxation benefits quickly, which further adds to the time efficiency for this age group (Akçayır et al., 2016). This is beneficial for a time-poor population such as young adults. Anderson et al. (2017) support that immersive VR is a feasible intervention for people that have time restrictions to aid relaxation.

People who engage in immersive virtual experiences tend to lose their sense of time (Sanders & Cairns, 2010) and feel that they are ‘relaxing for a lot longer than 15 minutes’. This could be interpreted through the theory of flow, in which a person is so engrossed in the activity at hand that their sense of time is altered (Csikszentmihalyi, 1975). When participants reported that the VR took them ‘to another place’, they were describing the concept of presence. Presence is a key concept of immersive VR and is described as a perceptual illusion that the user is actually present within the virtual environment and removed from their natural environment (Tham et al., 2018). A participant’s sense of presence is known to be enhanced by various features of the virtual environment, for example, realism of graphics and sounds and degree of immersion (Tham et al., 2018).

Participants reported that they were able to ‘escape everything that is going on’ while they were immersed in the VR experience. It has been shown that being able to distract yourself from a stress-inducing environment or situation helps to reduce stress levels (Villani et al., 2007). Using natural virtual environments shifts the participants’ attention away from the stressors that impact them, therefore, allowing for mental restoration when time restraints may prevent young adults from accessing real natural environments (Anderson et al., 2017).

Young adults are well versed in information technology, but paradoxically, this population demonstrates a limited understanding of the use of VR for relaxation within a health context. This may have led to many participants finding that the experience was *different than they thought it would be*. Most young adults are predominantly aware of VR use within the gaming context, which may have led them to enter this experience with preconceived ideas and expectations. There are numerous barriers to young adults utilising immersive VR for stress reduction such as the perception of high cost and lack of accessibility. This perception demonstrates the limited awareness of the evolving technology, which is becoming more affordable and accessible (Valmaggia et al., 2016). Despite this misunderstanding of VR due to preconceived ideas and barriers, all accounts were positive and exceeded expectations leading to a general consensus of the desire to access the technology again.

### Clinical implications

There is a preference of young adults for using technologies such as VR as a meaningful occupation in their life. This provides an opportunity in clinical practice for occupational therapists to use stress reduction VR programs. Whereby, occupational therapists can encourage young adults to broaden the use of VR from pure leisure to utilising it for management for stress. Furthermore, due to the mobility of the device and time-efficient nature of this technology, the implementation of one device could help many people to find relaxation during their periods of high stress. Implementation of VR technology by occupational therapists working in wellness programmes for workplaces may help to reduce the psychological stress of young adults, and in turn, contribute to a reduction in the poor mental health statistics for this age range. This may also simultaneously improve their productivity by helping to manage feelings of stress and increase their ability to engage in meaningful occupation. Specifically, this research identified that participants saw value in the use of VR in common spaces and break rooms within universities and workplaces. When implementing VR it must be taken into consideration that some people experience motion sickness, and this would not be suitable for them, despite this not occurring in this study. This research has expanded the evidence base for the use of immersive VR technology beyond clinical health care, providing preliminary evidence suggesting that it is equally beneficial for people without a mental health diagnosis. Additionally, this research expands the knowledge base and repertoire of clinical interventions for healthcare professionals and individuals to self-manage psychological stress.

### Limitations

The small sample size was moderated by researchers by using a mixed-method approach; however, the sample may still not be representative of young adults. Not knowing the exact resting heart rate data limits the establishment of accurate standard heart rate measurements. The reduction of the participants’ heart rates could have been caused by numerous other factors, such as the being seated and resting. The lack of a control group means that it was difficult to ascertain the exact causality of the results of the intervention.

Future research studies could expand on the number of participants recruited and elect to use a randomised control trial study design with an active control group such as mindfulness or meditation in the same dosage. The intervention itself could be extended over periods of days to weeks with additional heart rate measurements taken in order to establish better measures of heart rate. Measures could also be taken for additional biomarkers to heart rate. For example, blood pressure or saliva test to measure cortisol levels. Future studies could also recruit for different populations to determine if immersive VR is an effective tool for managing psychological stress across other age groups, in addition to young adults.

## Conclusion

The findings of this study suggest that immersive VR can reduce psychological stress in young adults. Individuals who participated in this study indicated that they experience high stress and that the immersive VR experience reduced their subjective and objective stress levels. They were able to achieve a calm state in a short amount of time. Engaging in the VR experience distracted them from their stresses and took them to another place. This experience challenged what young adults understood about VR and exceeded their expectations. This highlights the potential for this tool to be further explored as an intervention for managing stress levels in this age group.

**Key findings**

- Immersive VR is an acceptable method to assist young adults to manage psychological stress.
- Young adults perceived that immersive VR was a time-efficient strategy to reduce psychological stress.
- Immersive VR has the potential to be used widely to manage psychological stress of young adults.

**What the study has added**

This study adds to the evidence base that immersive VR can reduce perceived psychological stress, and it is a potential tool for occupational therapists working with young adults.
